# Piperazine-2,3,5,6-tetra­one

**DOI:** 10.1107/S1600536810048075

**Published:** 2010-11-27

**Authors:** Jing-Jing Jia, Xiu-Jin Meng, Shi-Zhang Liang, Shu-Hua Zhang, Yi-Min Jiang

**Affiliations:** a College of Chemistry and Chemical Engineering, Guangxi Normal University, Guilin, Guangxi 541004, People’s Republic of China; b College of Chemistry and Bioengineering, Guilin University of Technology, Guilin, Guangxi 541004, People’s Republic of China

## Abstract

The mol­ecule of the title compound, C_4_H_2_N_2_O_4_, is located around an inversion center and the four O atoms are in the 2,3,5,6-positions of the piperazine ring. In the crystal, bifurcated N—H⋯O hydrogen bonds link the mol­ecules into a corrugated layer parallel to (101).

## Related literature

For the synthesis of tetra­one, see: Norcross *et al.* (2008 ▶). For related structures, see Sletten *et al.* (1970 ▶, 1980 ▶); Sarangarajan *et al.* (2005 ▶); Norcross *et al.* (2008 ▶); Jin *et al.* (1998 ▶); Sanner *et al.* (1992 ▶); Ongania *et al.* (1985 ▶). 
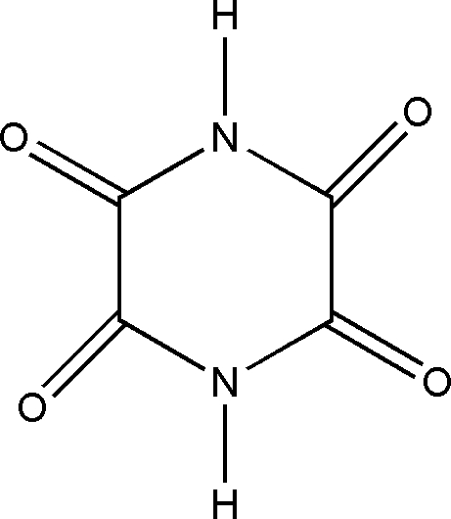


## Experimental

### 

#### Crystal data

C_4_H_2_N_2_O_4_
*M* *_r_* = 142.08
Monoclinic, 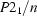

*a* = 5.163 (1) Å
*b* = 8.6220 (17) Å
*c* = 5.6540 (11) Å
β = 105.25 (3)°
*V* = 242.83 (8) Å^3^
*Z* = 2
Mo *K*α radiation
μ = 0.18 mm^−1^
*T* = 293 K
0.42 × 0.32 × 0.12 mm

#### Data collection

Siemens P4 diffractometer
Absorption correction: multi-scan (*SADABS*; Sheldrick, 1996 ▶) *T*_min_ = 0.930, *T*_max_ = 0.978
1357 measured reflections
438 independent reflections
383 reflections with *I* > 2σ(*I*)
*R*_int_ = 0.024

#### Refinement

*R*[*F* ^2^ > 2σ(*F* ^2^)] = 0.048
*wR*(*F* ^2^) = 0.092
*S* = 1.23
438 reflections
46 parameters
H-atom parameters constrained
Δρ_max_ = 0.20 e Å^−3^
Δρ_min_ = −0.21 e Å^−3^

### 

Data collection: *XSCANS* (Siemens, 1994 ▶); cell refinement: *XSCANS*; data reduction: *XSCANS*; program(s) used to solve structure: *SHELXS97* (Sheldrick, 2008 ▶); program(s) used to refine structure: *SHELXL97* (Sheldrick, 2008 ▶); molecular graphics: *ORTEPIII* (Burnett & Johnson, 1996 ▶), *ORTEP-3 for Windows* (Farrugia, 1997 ▶) and *PLATON* (Spek, 2009 ▶); software used to prepare material for publication: *SHELXTL* (Sheldrick, 2008 ▶).

## Supplementary Material

Crystal structure: contains datablocks I, global. DOI: 10.1107/S1600536810048075/dn2617sup1.cif

Structure factors: contains datablocks I. DOI: 10.1107/S1600536810048075/dn2617Isup2.hkl

Additional supplementary materials:  crystallographic information; 3D view; checkCIF report

## Figures and Tables

**Table 1:** Hydrogen-bond geometry (Å, °)

*D*—H⋯*A*	*D*—H	H⋯*A*	*D*⋯*A*	*D*—H⋯*A*
N1—H1⋯O1^i^	0.86	2.48	3.060 (2)	125
N1—H1⋯O2^ii^	0.86	2.23	3.035 (2)	157

Symmetry codes: (i) 
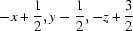
; (ii) 
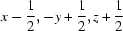
.

**Fig. 1.**
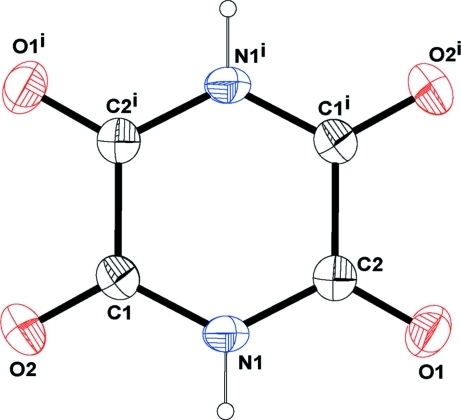
Molecular view of compound I with the atom labeling scheme. Ellipsoids are drawn at the 30% probability level. H atoms are represented as small spheres of arbitrary radii. [Symmetry code: (i) -x+1, -y+1, -z+1]

**Fig. 2.**
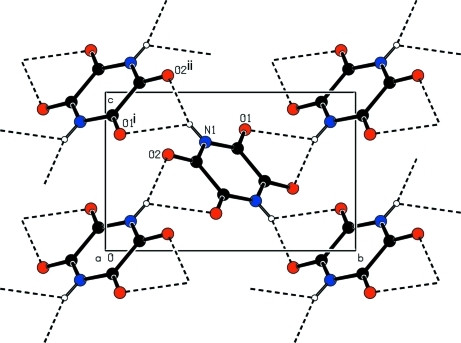
Partial packing view showing the corrugated layer parallel to the (1 0 1) plane. H bonds are shown as dashed lines. [Symmetry codes: (ii) -x+1/2, y-1/2, -z+3/2; (iii) x-1/2, -y+1/2, z+1/2]

## supplementary crystallographic information

### Comment

The synthesis and antitumor activity of some tetraone compounds have been widely studied (Jin *et al.*, 1998; Sanner *et al.*, 1992). Most tetraone compounds were found from a naturally occurring alkaloid in a variety of leguminous plant and tree species, including broom, lupin, gorse, and laburnum(Norcross *et al.*, 2008). As part of our interest in the synthesis of tetraone derivatives, we report here the structure of the title compound.

The molecule of the title compound is located around inversion center and the four O atoms are in the 2,3,5,6 position on the piperazine ring (Fig. 1). The molecule is planar with rms deviation of 0.013Å. The bond distances and angles are similar to those found in related piperazine derivatives (Sletten *et al.*, 1970; Sarangarajan *et al.*, 2005; Sletten *et al.*, 1980; Ongania *et al.*, 1985).

The N—H donor and the C—O acceptor groups participate in the hydrogen bonding forming corrugated layers parallel to the (1 0 1) plane through bifurcated N-H···O hydrogen bonds (Table 1, Fig. 2).

### Experimental

For the preparation of the title compound,the 2-mercaptopyrazine (10 mmol,1.1200 g) was dissolved in ethanol (50 ml) at 358 K and a solution of 30% H_2_0_2_ (10 ml) was added. The resulting solution was stirred at 358 K for 4 h, then concentrating at 388 K,until 3 ml solution remained. Colourless-block crystal suitable for X-ray diffraction were obtained by slow evaporation at room temperature after several days in 55% yield.

### Refinement

H atom attached to N atom was positioned geometrically and treated as riding on the parent atom with N-H= 0.86 Å and *U*_iso_(H)=1.2*U*_eq_(N).

### Crystal data

**Table:**

C_4_H_2_N_2_O_4_	*F*(000) = 144
*M**_r_* = 142.08	*D*_x_ = 1.943 Mg m^−^^3^
Monoclinic, *P*2_1_/*n*	Mo *K*α radiation, λ = 0.71073 Å
Hall symbol: -P 2yn	Cell parameters from 438 reflections
*a* = 5.163 (1) Å	θ = 4.4–25.3°
*b* = 8.6220 (17) Å	µ = 0.18 mm^−^^1^
*c* = 5.6540 (11) Å	*T* = 293 K
β = 105.25 (3)°	Block, colourless
*V* = 242.83 (8) Å^3^	0.42 × 0.32 × 0.12 mm
*Z* = 2	

### Data collection

**Table:**

Siemens P4 diffractometer	438 independent reflections
Radiation source: fine-focus sealed tube	383 reflections with *I* > 2σ(*I*)
graphite	*R*_int_ = 0.024
ω scans	θ_max_ = 25.3°, θ_min_ = 4.4°
Absorption correction: multi-scan (*SADABS*; Sheldrick, 1996)	*h* = −6→6
*T*_min_ = 0.930, *T*_max_ = 0.978	*k* = −10→10
1357 measured reflections	*l* = −6→6

### Refinement

**Table:**

Refinement on *F*^2^	Primary atom site location: structure-invariant direct methods
Least-squares matrix: full	Secondary atom site location: difference Fourier map
*R*[*F*^2^ > 2σ(*F*^2^)] = 0.048	Hydrogen site location: inferred from neighbouring sites
*wR*(*F*^2^) = 0.092	H-atom parameters constrained
*S* = 1.23	*w* = 1/[σ^2^(*F*_o_^2^) + (0.0355*P*)^2^ + 0.1147*P*] where *P* = (*F*_o_^2^ + 2*F*_c_^2^)/3
438 reflections	(Δ/σ)_max_ < 0.001
46 parameters	Δρ_max_ = 0.20 e Å^−^^3^
0 restraints	Δρ_min_ = −0.21 e Å^−^^3^

### Special details

**Table:**

Geometry. All esds (except the esd in the dihedral angle between two l.s. planes) are estimated using the full covariance matrix. The cell esds are taken into account individually in the estimation of esds in distances, angles and torsion angles; correlations between esds in cell parameters are only used when they are defined by crystal symmetry. An approximate (isotropic) treatment of cell esds is used for estimating esds involving l.s. planes.
Refinement. Refinement of *F*^2^ against ALL reflections. The weighted *R*-factor *wR* and goodness of fit *S* are based on *F*^2^, conventional *R*-factors *R* are based on *F*, with *F* set to zero for negative *F*^2^. The threshold expression of *F*^2^ > σ(*F*^2^) is used only for calculating *R*-factors(gt) *etc*. and is not relevant to the choice of reflections for refinement. *R*-factors based on *F*^2^ are statistically about twice as large as those based on *F*, and *R*- factors based on ALL data will be even larger.

### Fractional atomic coordinates and isotropic or equivalent isotropic displacement parameters (Å^2^)

**Table:**

	*x*	*y*	*z*	*U*_iso_*/*U*_eq_	
O1	0.1613 (3)	0.55814 (19)	0.7646 (3)	0.0343 (5)	
O2	0.8069 (3)	0.25100 (19)	0.6060 (3)	0.0342 (5)	
N1	0.4775 (4)	0.3995 (2)	0.6856 (3)	0.0261 (5)	
H1	0.4603	0.3362	0.7981	0.031*	
C1	0.6621 (5)	0.3635 (2)	0.5614 (4)	0.0233 (5)	
C2	0.3169 (4)	0.5281 (2)	0.6461 (4)	0.0233 (5)	

### Atomic displacement parameters (Å^2^)

**Table:**

	*U*^11^	*U*^22^	*U*^33^	*U*^12^	*U*^13^	*U*^23^
O1	0.0357 (10)	0.0355 (10)	0.0373 (10)	0.0010 (8)	0.0197 (9)	−0.0034 (8)
O2	0.0348 (10)	0.0268 (9)	0.0402 (10)	0.0099 (8)	0.0086 (8)	0.0048 (7)
N1	0.0330 (11)	0.0229 (10)	0.0248 (11)	0.0004 (9)	0.0120 (9)	0.0053 (8)
C1	0.0221 (12)	0.0205 (11)	0.0256 (12)	−0.0019 (10)	0.0034 (10)	−0.0017 (9)
C2	0.0224 (12)	0.0225 (11)	0.0239 (12)	−0.0027 (10)	0.0042 (10)	−0.0038 (9)

### Geometric parameters (Å, °)

**Table:**

O1—C2	1.202 (3)	N1—C2	1.368 (3)
O2—C1	1.210 (3)	N1—H1	0.8600
N1—C1	1.360 (3)	C1—C2^i^	1.526 (3)
			
C1—N1—C2	125.31 (19)	N1—C1—C2^i^	117.28 (19)
C1—N1—H1	117.3	O1—C2—N1	123.3 (2)
C2—N1—H1	117.3	O1—C2—C1^i^	119.3 (2)
O2—C1—N1	123.6 (2)	N1—C2—C1^i^	117.35 (18)
O2—C1—C2^i^	119.10 (19)		

Symmetry codes: (i) −*x*+1, −*y*+1, −*z*+1.

### Hydrogen-bond geometry (Å, °)

**Table:**

*D*—H···*A*	*D*—H	H···*A*	*D*···*A*	*D*—H···*A*
N1—H1···O1^ii^	0.86	2.48	3.060 (2)	125
N1—H1···O2^iii^	0.86	2.23	3.035 (2)	157

Symmetry codes: (ii) −*x*+1/2, *y*−1/2, −*z*+3/2; (iii) *x*−1/2, −*y*+1/2, *z*+1/2.
