# Piperazine-2,3,5,6-tetra­one. Corrigendum

**DOI:** 10.1107/S1600536810052712

**Published:** 2010-12-18

**Authors:** Jing-Jing Jia, Xiu-Jin Meng, Shi-Zhang Liang, Shu-Hua Zhang, Yi-Min Jiang

**Affiliations:** a College of Chemistry and Chemical Engineering, Guangxi Normal University, Guilin, Guangxi 541004, People’s Republic of China; b College of Chemistry and Bioengineering, Guilin University of Technology, Guilin, Guangxi 541004, People’s Republic of China

## Abstract

The correspondence author in the paper by Jia *et al.* [*Acta Cryst.* (2010), E**66**, o3315] is corrected.

## Abstract 2

Corrigendum to *Acta Cryst.* (2010), E**66**, o3315.

In the paper by Jia *et al.* (2010) ▶, the correspondence author is incorrectly given as ‘Jing-Jing Jia’. The correct correspondence author is ‘Yi-Min Jiang’, as given above.
